# Instant Clue: A Software Suite for Interactive Data Visualization and Analysis

**DOI:** 10.1038/s41598-018-31154-6

**Published:** 2018-08-23

**Authors:** Hendrik Nolte, Thomas D. MacVicar, Frederik Tellkamp, Marcus Krüger

**Affiliations:** 1 Institute for Genetics and Cologne Excellence Cluster on Cellular Stress Responses in Aging-Associated Diseases (CECAD), University of Cologne, Joseph-Stelzmann-Strasse 26, 50931 Cologne, Germany; 2 Department of Dermatology, Center for Molecular Medicine Cologne, University of Cologne, 50931 Cologne, Germany; 3 Center for Molecular Medicine (CMMC), University of Cologne, 50931 Cologne, Germany

## Abstract

The development of modern high-throughput instrumentation and improved core facility infrastructures leads to an accumulation of large amounts of scientific data. However, for a majority of scientists the comprehensive analysis and visualization of their data goes beyond their expertise. To reduce this hurdle, we developed a software suite called Instant Clue that helps scientists to visually analyze data and to gain insights into biological processes from their high-dimensional dataset. Instant Clue combines the power of visual and statistical analytics using a straight forward drag & drop approach making the software highly intuitive. Additionally, it offers a comprehensive portfolio of statistical tools for systematic analysis such as dimensional reduction, (un)-supervised learning, clustering, multi-block (omics) integration and curve fitting. Charts can be combined with high flexibility into a main figure template for direct usage in scientific publications. Even though Instant Clue was developed with the omics-sciences in mind, users can analyze any kind of data from low to high dimensional data sets. The open-source software is available for Windows and Mac OS (http://www.instantclue.uni-koeln.de) and is accompanied by a detailed video tutorial series.

## Introduction

The development of fast and robust instruments for unbiased high-throughput experiments allows scientists to routinely generate large-scale datasets. Numerous techniques to generate omics-data have reached a level that makes them broadly applicable and powerful technologies for medical and biological researchers. For example, with the latest generation of mass spectrometry instruments, it is possible to quantify a global proteome of more than 12,000 proteins within a day. In addition, the enrichment of post-translational modifications, including phosphorylations and ubiquitylations results in more than 40,000 modification sites, that can be achieved with moderate laboratory effort^1–4^. Such experiments demand cooperative work between biologists with specialized knowledge in their field and computational researchers to unravel meaningful findings. In many cases the standard statistical analysis such as the identification of differentially expressed genes, metabolites or proteins is performed by a dedicated bioinformatics core unit or via easy-to-use desktop^5–7^ or web interface tools^8^ that have been developed to partly overcome this problem. However, the visualization, exploration and statistical analysis of these datasets can still be challenging for a majority of biological or medical researchers.

Here, we address this gap by developing a software suite that enables scientists independent of their computational background to analyze their own complex data. We aimed to develop a tool fulfilling the following challenges: (i) Applicability to a broad range of data inputs from various experiments like densitometric data from immunoblots, quantitative PCR, high-dimensional molecular data sets like proteomic or transcriptomic data, as well as complex time series data. (ii) Highly intuitive data analysis and visualization by drag & drop that allows new users to rapidly draw conclusions from their data. The underlying concept and Graphical User Interface (GUI) design has been inspired by the software Tableau/Polaris^9^. (iii) Offering a repertoire of statistical tests like ANOVA, regressions, curve fitting and principal component analysis as well as facilitating calculation of metrics like area under the curve (AUC) in an interactive way. Importantly, the application of a statistical test requires the visual inspection of the data by the user. This workflow was implemented to help the user to interpret the test results. (iv) Availability of functions to filter, annotate and select data from complex datasets. (v) Facilitate the collection of charts to generate figures with little post-processing prior to publication.

To address these aims, we have created a software suite called Instant Clue which is available for Mac OS and Windows at http://www.instantclue.uni-koeln.de including detailed tutorials and working examples. In addition, the python source code is also freely available making Instant Clue editable and available on any platform with a working Python version (>=3.4).

## Methods

### Software design

We took advantage of the constantly growing number of scientific packages in Python and wrote Instant Clue in pure Python (>3.4). Moreover, Python is known for its design philosophy that emphasizes code readability and user specific adjustments can be achieved by a broad range of researchers. The presented software is open-source and relies mainly on the following packages: tkinter for the graphical user interface, pandas and pandastable for data management^10,11^, matplotlib^12^ and seaborn^13^ for generating charts, numpy^14^ for calculations as well as statsmodels^15^, scikit-learn^16^, and Cython^17^ for statistical tests. The Graphical User Interface (GUI) (Fig. S1) is built with python’s standard tool kit tkinter/tkk. The GUI represents a scaffold to use the implement library modules which are grouped by their function. For example, there is a module to provide easy data management and manipulations (data.py) separated from the plotter module which handles plotting events (plotter.py). The general software architecture is illustrated in Fig. S2.

### Download and Maintenance

The software can be downloaded from http://www.instantclue.uni-koeln.de for Windows and Mac OS as a zip file. Mac OS users need to install Active Tcl Version 8.5.18 before usage (https://www.activestate.com/activetcl/downloads). Instant Clue will be updated continuously and users are alerted upon new version releases. Support for users is provided by our detailed tutorial series at http://www.instantclue.uni-koeln.de/tutorials.html in written and commented video format.

### Bug Report and Feature Requests

Users that detect unexpected behavior or missing features are highly encouraged to report this to us via GitHub (https://github.com/hnolCol/instantclue/issues) or direct mail contact.

### Example Data

To facilitate a comfortable start for users, we have included several example data sets that can be found in the compressed file (folder: examples). In light of the versatile application of Instant Clue, we have included fully documented step-by-step data analysis procedure in the tutorial (http://www.instantclue.uni-koeln.de/tutorials.html) of various different type of data sets: (i) Body weight measurements of people of different health condition and age. (ii) mass spectrometry based immunoprecipitation data published recently to identify interaction partners of a protease dead mutant of Presenilins-associated rhomboid-like protein (PARL)^18,19^. (iii) optical recording of Pro-opiomelanocortin (POMC) neuron activity (time series data)^20^. (iv) iris data set^21^. (v) wine quality data set for supervised learning^22^. As the tutorial will be extended continuously, we will also add more example data.

## Results and Discussion

Throughout the software, the general concept is that the analysis of data is driven by visual inspection. Thus, we employed drag & drop events as the central mean of action to plot charts as well as to apply statistical tests and techniques. This enforces the user to inspect data visually, which will help to interpret, verify and judge results. In the following, we will explain general aspects and give an overview of the presented software. In addition, we have uploaded several video tutorials that will support users to become familiar with Instant Clue (http://www.instantclue.uni-koeln.de/videos.html). In Instant Clue all activities are initiated via the Graphical User Interface (GUI) that is explained in Figs S1 and S2.

### Data organization and plotting

**Figure 1:**
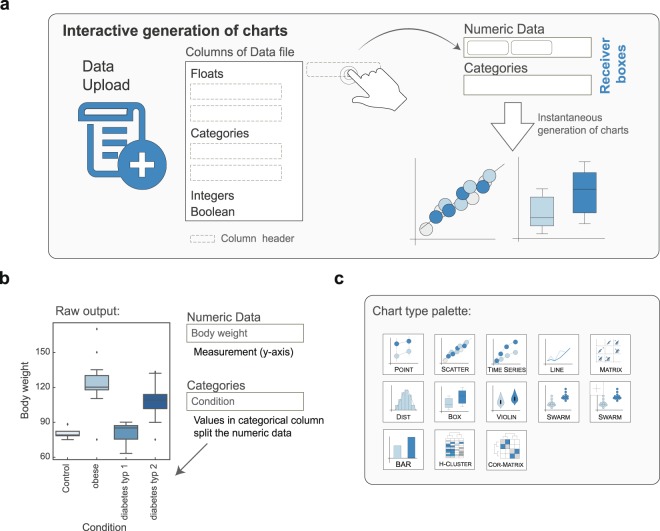
Instant Clue allows on-the-fly inspection of data. (**a**) Schematic workflow of interactive chart generation by drag & drop. Upon data-upload the column headers are displayed in a tree-like view and separated according to their data type. The two receiver boxes can accept column headers and trigger data visualization. (**b**) Raw output of two drag & drop steps. The displayed data are from the accompanied Tutorial_Data_1.xslx file that can be found in the install directory. (**c**) Available chart type palette.

Data can be uploaded from various file types including Excel, tab delimited text (.txt) and csv-files (.csv), Extensible Markup Language (.xml) files as well as compressed files (.gz, .zip). Once uploaded the data columns are automatically separated by their datatype. The four available datatypes are Numeric Floats (example: 1.345), Integers (1, 1922), Categories (Time, Genotype, Gene names) and Boolean (True, False) (Fig. 1a). Because several functions require certain data types, the type of a column can be changed retroactively. Users might also upload several files that can be merged.

Charts are generated instantaneously by drag & drop of column headers to one of the two receiver boxes (Figs 1a and S1). The categorical box is used to split data according to the present categories. For example, numeric data represent measurements such as body weight or gene expression values. Categorical columns contain categorical values such as the state of a disease, genotype or experimental setup (treatment, no treatment). As an example, Fig. 1b depicts the raw on-the-fly output of Instant Clue after loading the accompanied “Tutorial_Data_01.xlsx” and adding the Body weight column to the numerical data receiver box and the Condition column to the categorical receiver box by drag & drop. The chart type can be chosen from numerous available options, each of which is specialized for a certain type of data and way of visualization (Fig. 1c). Advantages of each chart type are summarized in the online tutorial. Users can easily modify chart margins, font size and axis limits in an interactive way and export charts to numerous file types.

### Computational activities and data filtering

Instant Clue offers a diverse portfolio of computational activities to assist the visual exploration of multivariate data. Activities are applied on columns in the dataset using the context menu and cover basic steps such as sorting, string splitting and replacement, normalization and transformation, imputation of missing values, smoothing and rolling window calculations. Additionally, the data format can be changed between long and wide formats and numerous calculations such as Z-Score, mean and standard deviation row-wise or kernel density estimations column-wise are implemented. A detailed description of each activity is presented in the pdf tutorial at http://www.instantclue.uni-koeln.de/tutorials.html.

**Figure 2:**
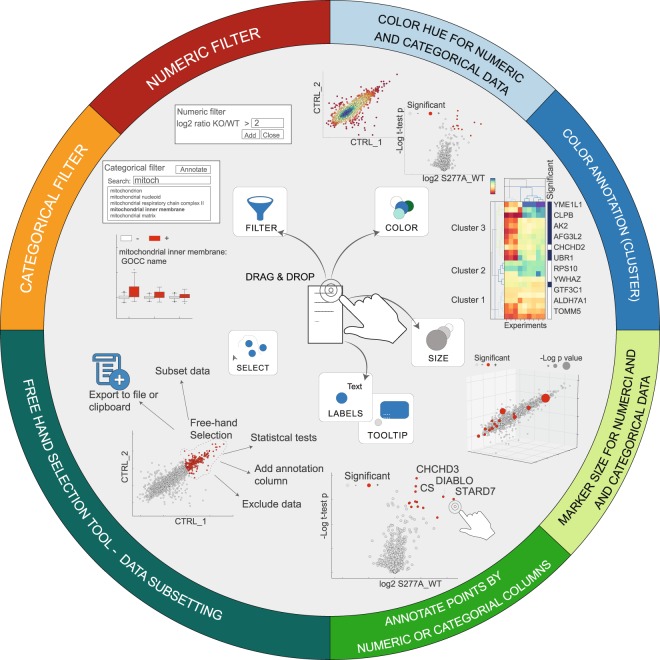
Data Filtering and extending a chart’s information content. Description clockwise starting from top right. The color button allows not only to change the color of an existing graph but also to encode categorical or numerical values by color. For charts such as scatter, principal component driver or line plot the direct chart elements are color encoded. For hierarchical clustering an additional color column is added. Similar to the color button, size can be used as another level of information content in a chart. Scatter points can be annotated by selecting a column for the label button. The tooltip button can be used to hover over chart items to quickly screen through the data (*bottom-right*). The selection tool allows the user to select data in a scatter plot for sub setting and further analysis (*bottom-left*). The filter button can be used to quickly apply categorical and numerical filtering (*top-left*).

To systematically evaluate differences between biological samples, researchers aim to subset their data by certain criteria such as cellular localization or signaling pathway based on annotation terms derived from several sources such as the gene ontology^23^, GSEA^24^, MitoCarta^25^ or PFAM^26^ database. Therefore, we have implemented numerous categorical filters that allow for quick but complex filtering. There are three different categorical filters: (i) ‘Find Category & Annotate’, (ii) ‘Find String(s) & Annotate’ and (iii) ‘Custom Categorical Filter’. A summary of all filtering steps, advantages and example results are displayed in Fig. S3. To visualize applied filters, the “Slice and Marks Frame” option (Fig. S1) allows for color/size encoding via Drag & Drop that might be used to highlight significantly different expressed proteins or genes (Fig. 2 top-right). The tooltip and label activities (Fig. 2 bottom) facilitate fast and efficient screening through the dataset. For instance, these activities can be used to enable the annotation and identification of interesting candidates in a scatter plot or hierarchical clustering.

### Instant Clue comprises a Statistical Toolbox for multivariate data analysis

**Figure 3:**
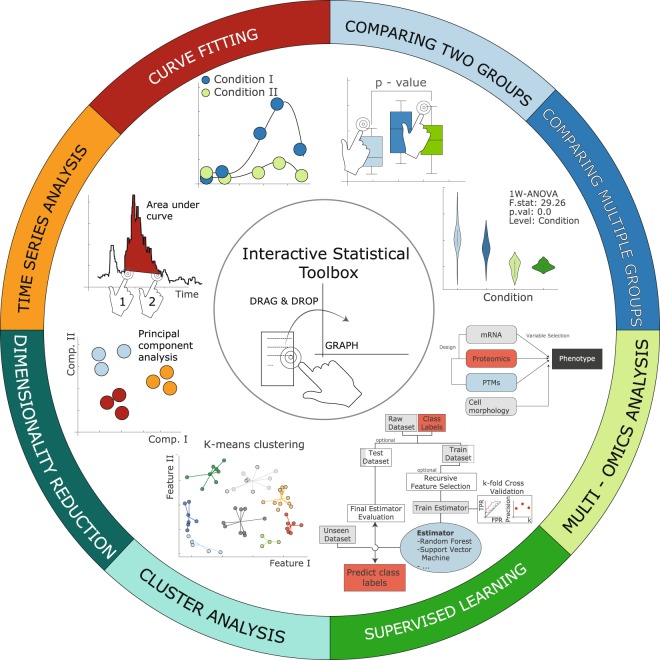
Statistical Toolbox. Description clockwise starting from top-right. The statistical toolbox offers functionality to compare two-groups using non-parametric and parametric tests (e.g. t- and U-test) or multiple groups (ANOVA, Kruskal Wallis). Up to a three-way ANOVA design is available with and without repeated measurements (*top-right*). To integrate multiple omics data, users can use method such as sparse generalized canonical correlation analysis (SGCCA)^35^ to select features such as genes, proteins, miRNAs that strongly contribute to the multi-omics signature (*right*). A comprehensive toolbox for supervised learning has been implemented using the scikit-learn library allowing the user to set up pipelines with data-preprocessing, feature selection and prediction (*bottom*). Moreover, principal component analysis and cluster analysis can be initialized by one simple drag & drop event. Time series data can also be explored and techniques such as base line correction and measuring the area under curve (AUC) are implemented in an interactive way. (*top-left*) Row-wise curve fitting as well as correlation-based analysis can be performed in a customizable and versatile way. Due to its diversity and complexity all functions cannot be described here and further information and examples can be found at: https://www.instantclue.uni-koeln.de.

Instant Clue promotes the visual analysis of data, but also offers several statistical tests that are applied in an interactive way. In line with the idea that researchers should inspect data visually as a first step, statistical tests are enabled by a drag & drop event from the Analysis toolbox onto generated charts. Several tests are automatically performed and do not require further action by the user. Nevertheless, for comparing two groups via t-test or U-test, the statistical assessment is only enabled after a drag & drop action. By clicking on the desired groups that should be compared, the test is automatically calculated, and the p-value is indicated in the chart above lines between tested groups (Fig. 3 – top right). In addition, performed tests are stored and can be exported at any time. Noteworthy, if an activity (each test is an activity) cannot handle missing values, the data are automatically filtered before submission to the specified activity, without changing the source data. The toolbox covers numerous techniques, including supervised learning, clustering, dimensional reduction, time series as well as curve fitting. In the following we describe and present results of supervised learning, time series and curve fitting to illustrate the functionality and ease of the presented software.

### (Un-)Supervised Learning for data classification

The ability to generate high dimensional data with moderate effort and depth as well as the massively increasing knowledge in science facilitates the application of supervised learning techniques. In general, these methods are utilized to predict class memberships based on a learning process. In this step a training dataset is used to build inferred function that is used to classify new unseen samples. The training dataset consist of *n* samples and *m* features as well as the class labels. For example, a training dataset could encompass several thousand human subjects (samples) that were screened in a hospital measuring several parameters (features) such as blood pressure, weight, or the number/location of single nucleotide polymorphisms (SNPs) found in the genome, describing the subject’s health condition (class labels – healthy, cancerous). These data can be used to train an estimator which in turn is able to predict a health condition based on the used features for uncharacterized subjects. Such classification tasks were successfully used to predict new kinase-substrate relationships^3^ and many other applications in biological and medical science^27,28^. Instant Clue offers several functions to establish an estimator for prediction, based on the scikit-learn library^16^. Users can optimize pre-processing of data, feature selection/reduction and estimator parameters using exhaustive grid searching over given parameters. Fig. S4 shows the dialog window to interactively construct a prediction pipeline. A pre-processing step might be used to scale/normalize the input data. To increase the generalization ability, accuracy and prediction speed of an estimator it is often useful to select the most important features or to apply a dimensional reduction technique (feature selection) before training an estimator. These steps can be defined using an interactive drag & drop dialog window (Fig. S4). Established pipelines can be saved and subsequently used to predict class memberships of unseen data. Thus, Instant Clue provides a convenient way to accomplish classification tasks.

Moreover, the software offers several functions to analyze data in an unsupervised fashion such as Principal Component Analysis (PCA), k-means or Density-based spatial clustering of applications with noise (DBSCAN) clustering allowing users to identify underlying patterns. Fig. S5 illustrates the raw output of a PCA and k-means clustering analysis. Clustering algorithms can also be utilized to predict cluster membership of unseen data.

### Time series analysis

Instant Clue offers the possibility to explore time series data. The software is currently limited to continuous time data such as increasing number of seconds/minutes. The activities to analyze time series data aim to smooth data such as an intensity along a time axis. The Example Data 03 (see Methods) are optical recordings of Pro-opiomelanocortin (POMC) neuron activity. Signal measurements over time can be baseline corrected and the area under curve (AUC) can be determined in an interactive way. (Fig. 3 top-left and Fig. S6). Noteworthy, even though these activities are limited to the time series chart type (Fig. 1c) the x axis can be any continuous data array such as m/z or scan number.

### Curve fitting and correlation analysis

Curve fitting and correlation is an efficient way to connect phenotype characterizing data such as blood glucose levels, body mass index, blood pressure or fitness to expression data of proteomic or genomic experiments. This fundamental principle was first discovered by Linus Pauling in 1940, when he observed that a single amino acid change in Hemoglobin caused a structural change of the protein, which eventually results in the development of sickle cell anemia^29^. Today, scientists are able to create causal networks on a more comprehensive scale, mostly driven by correlation analysis. It has recently been demonstrated how three distinct omics levels provide in-depth insights into the molecular mechanisms and how they correlate to the characterized phenotype^30^. Therefore, we have added a toolbox to perform curve fitting and correlation analysis in an intuitive way. Several functions are implemented such as polynomial or linear fits, enzymatic reaction models (Michaelis-Menten), and periodic functions to identify genes/proteins that are following circadian rhythm (Fig. S7).

### The Main Figure Template promotes structured collection of charts

**Figure 4:**
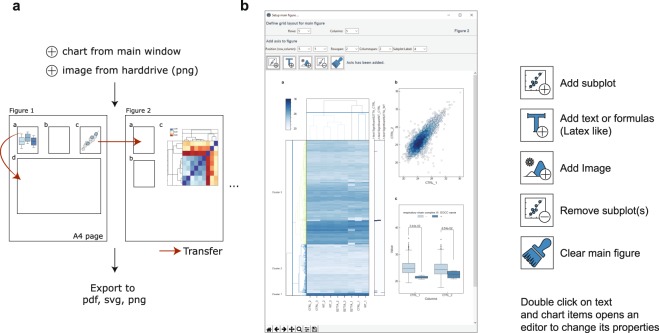
Collecting charts in a main figure template. (**a**) Workflow to create a main figure template consists of three steps that allows the collection of charts made in Instant Clue or from the user’s documents (Portable Network Graphics (png) files). (i) Create a main figure of fixed size (A4 paper). (ii) add subplot at desired position and size. The size of a subplot is determined by the underlying grid (more information can be found at http://www.instantclue.uni-koeln.de/videos.html – commented video tutorial). Transfer figure from Instant Clue’s main window to the created subplot. Exported charts can be moved to other subplots also in another main figure template. (**b**) Example screenshot of a main figure that consists of three subplots with varying sizes. Several activities are available to modify main figures such as: adding subplots, text or images. Notably, elements of a chart such as the x-axis label, the color of a bar or annotation’s text size can be edited in the main figure. Conveniently, the font of text elements or capitalization of subplot labels can be performed on all created main figures simultaneously facilitating the adjustment to journal requirements. Data were taken from the provided Example 2 that was recently published^18,19^.

Scientists often seek to integrate several charts into figures containing multiple subplots. Even though plots can be easily exported as vector graphics directly from the main window which can be further processed in suitable vector graphic software tools. In addition, we also provide the possibility to combine several charts and images in so-called main figure templates (Fig. 4). To this end, we have generated activities to: (i) create multiple main figure templates (ii) add labeled subplots to these figure templates (iii) incorporate charts from Instant Clue’s main window or for adding image files from the user’s documents (Fig. 4a). Users can delete, move and modify elements of a chart, define subplot labels and add text or formulas resulting in a publication-ready figure without further software tools (Fig. 4b). In practice, the main figure template ensures the same format between subplots, helps to generate an uniform figure presentations, and clearly reduces the processing time. Main figures can be exported to numerous file types including pdf, svg or png.

### Comparison with other tools

To highlight Instant Clue’s advantages and the contribution to the field, we have compared the presented tool with other published and free of charge software suites (Table 1). Each software tool has its own strengths and weaknesses since they were developed to address different needs of users. Instant Clue’s functionality covers a unique variety of scientific tools from time series analysis over curve fitting to multi-omics data analysis. In addition, Instant Clue aims to combine a rich statistical toolbox with visualization that are suitable for usage in scientific publications with little post-processing. While sophisticated tools such as KNIME^31^, Orange^32^, Voyager 2^33^ and GProX^34^ offer functionality for data analysis and visualization with overlap to the presented tool, Instant Clue has a unique and wide-ranging combination of features within one software suite: (i) interactive live filtering and masking of data (ii) broadly applicable analysis and visualization activities used in various fields of life sciences and therefore broadly applicable (iii) comprehensive categorical filtering to subset and annotate data without a single line of code (iv) highly adjustable charts such as annotated text labels and (v) intuitive main figure templates to collect multiple charts for publication with minimal post-processing. However, software such as Orange and KNIME are based on constructing pipelines for data analysis with great overview and applicability to other data sets which is currently not implemented in Instant Clue. Tools that are specialized on visual analytics such as Voyager 2 include a sophisticated algorithm that infers graphical representation, which might help the user to gain more insights into their data. Though not as advanced, we have implemented algorithms in Instant Clue that infer a graphical representation that are commonly used in life science data plotting. Overall, we are confident that the unique combination of a comprehensive statistical toolbox, interactive and dynamic live filtering, and flexibility in chart generation offered by Instant Clue will be a helpful and complementary approach for scientists from interdisciplinary areas to analyze complex data sets.

**Table 1:** Features and Requirements of the current plethora of tools with overlap to Instant Clue’s functionality.

Name	Open Source	Platform Independent	Main Focus	Graph Suggestions	Workflow (History)	Interactive data filtering	Main Figure Template	Ref.
GProX	+	−	Proteomics	−	−	+	−	^34^
Instant Clue	+	+	General/Omics	+	− (b)	+++	+	This study
KNIME	+	+	General	−	++	++	−	^31^
Orange	+	+	Machine Learning/Visualization	−	++	++	−	^32^
Perseus	− (parts)	−	−omics data	−	+	+	−	^5^
Voyager 2/Vega	+	++ (a)	Visual analytics	+++	−	+++	−	^33^

The statistical language R or Python packages such as matplotlib or plotly offer immense flexibility but require programming skills and are therefore not included  above. Moreover, proprietary software tools are excluded. Tools are presented in alphabetical order. Note in table: a – web service, b – incorporated in the current developmental version.

## Conclusion

The routine generation of high dimensional datasets demands the cooperative work between bioinformaticist and biological researcher. To equip scientists that are faced by the challenge of visualizing and analyzing multifactorial data with a straightforward tool, we have developed Instant Clue. Due to its simplicity, attractive design and intuitive drag & drop interface, the software can assist in the fast and comprehensive analysis of various datasets. The wide-ranging functionality of Instant Clue covers numerous charts, however we are aiming to extend the panel of statistical tests and will add further activities that will be beneficial for systematic data interpretation.

Moreover, advanced users are not limited to the portfolio of activities and can modify the source code to adjust the software to their needs. We encourage computer experts to contribute to the development of Instant Clue, sharing their adjustments with us, and thereby accelerate the continuous improvement process. We are confident that this software will facilitate the communication between interdisciplinary scientists.

## Electronic supplementary material

Supporting Information
