# Supplementary material for: Instant Clue: A Software Suite for Interactive Data Visualization and Analysis
Source: Sci Rep. 2018 Aug 23;8:12648. doi: 10.1038/s41598-018-31154-6 (PMC6107636; doi:10.1038/s41598-018-31154-6)
Supplement: Supplementary file 1 — Supporting Information [file 41598_2018_31154_MOESM1_ESM.docx]

Instant Clue: A Software Suite for Interactive Data Visualization and Analysis

Hendrik Nolte, Thomas D. MacVicar, Frederik Tellkamp and Marcus Krüger

Institute for Genetics and Cologne Excellence Cluster on Cellular Stress Responses in Aging-Associated Diseases (CECAD), University of Cologne, Joseph-Stelzmann-Strasse 26, 50931 Cologne, Germany.

**SUPPLEMENTARY INFORMATION**

**CONTENT**

Figure S1 – Graphical User Interface (GUI).

Figure S2 - Architecture of Instant Clue.

Figure S3 - Categorical Filtering in Instant Clue.

Figure S4 – Supervised learning pipeline

Figure S5 – Principal Component Analysis and k-means clustering.

Figure S6 – Time series analysis

Figure S7 – Curve fitting.

**
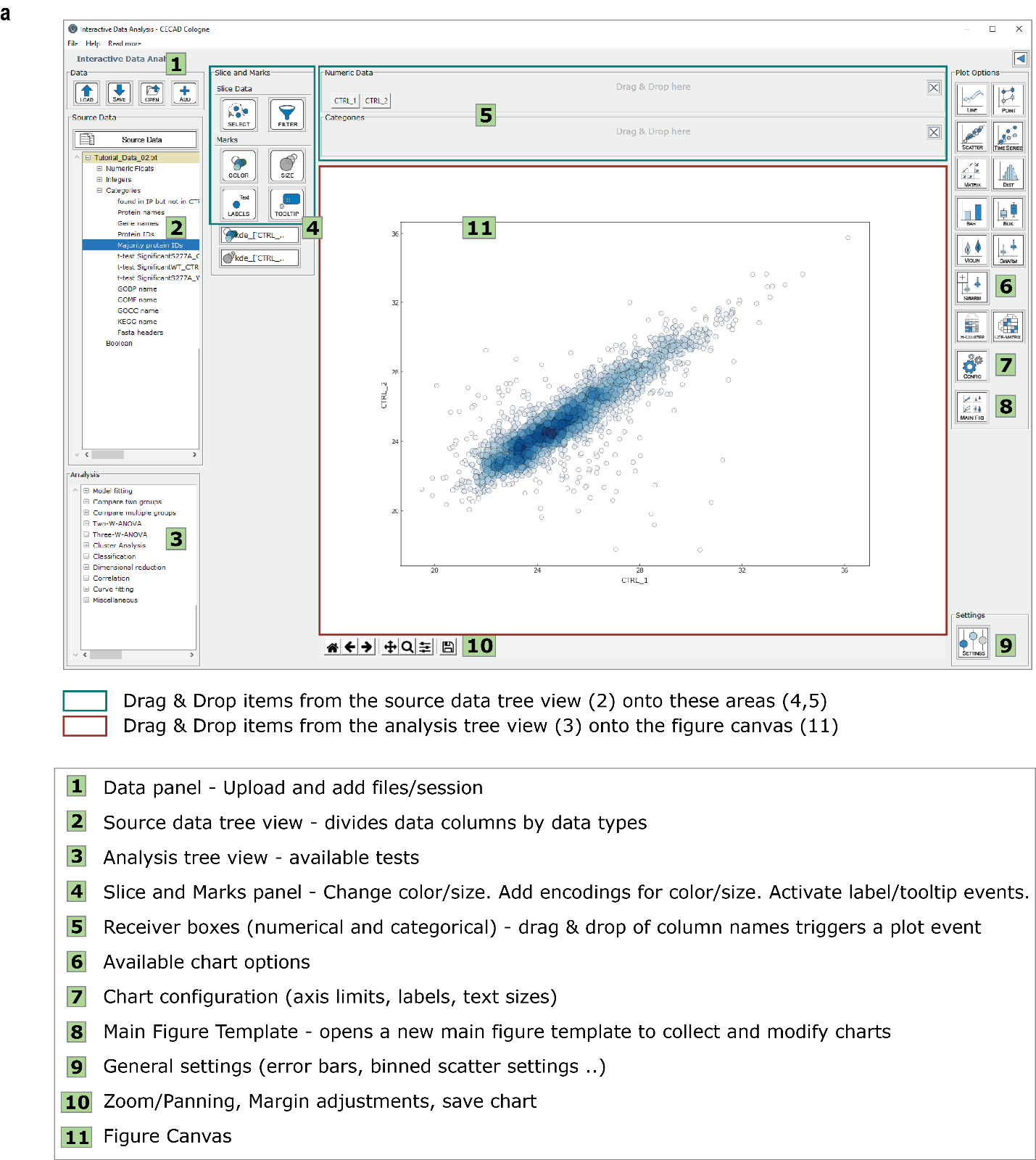
**

**Figure S1 – Graphical User Interface (GUI).** Screenshot using Windows 10 showing the different parts of the main window. 1 – Data frame to upload and add data from numerous file types (Load, Add). Sessions can be saved using the save button and uploaded using the open button. 2 – Source data tree view – divides the column headers of an uploaded file by their data types (Floats, Integers, Categories, Boolean). 3 – Analysis tree view – displays available statistical activities. 4 – Slice and marks – all icons (except the “selection” icon) can receive columns headers from the source data tree view (2) to color/size code a chart or to annotate specific or screen through interesting scatter points. 5 – Numerical and categorical receiver boxes – accept drag & drop events from the source data tree view that will trigger a plot event. 6 – Chart palette. 7 – Configuration: opens a dialog window to modify chart settings such as axes labels, text sizes and axis limits. 8 – Main Figure Template: Opens a new main figure window to collect/modify charts. 9 – General settings opens a dialog window to modify settings such as the method used for hierarchical clustering, dimensional reduction or what error bars should represent. 10 – Toolbar to modify margins, zoom and pan or to save a figure. Mac users will need to specify the file type by changing the file name’s extension (*.png - *.pdf). 11 – Figure canvas. To apply a test from the analysis tree view (3) the user needs to select the test and drag & drop it onto the figure canvas (red border). This follows the idea that users have to explore their data visually before a statistical test is applied.

**
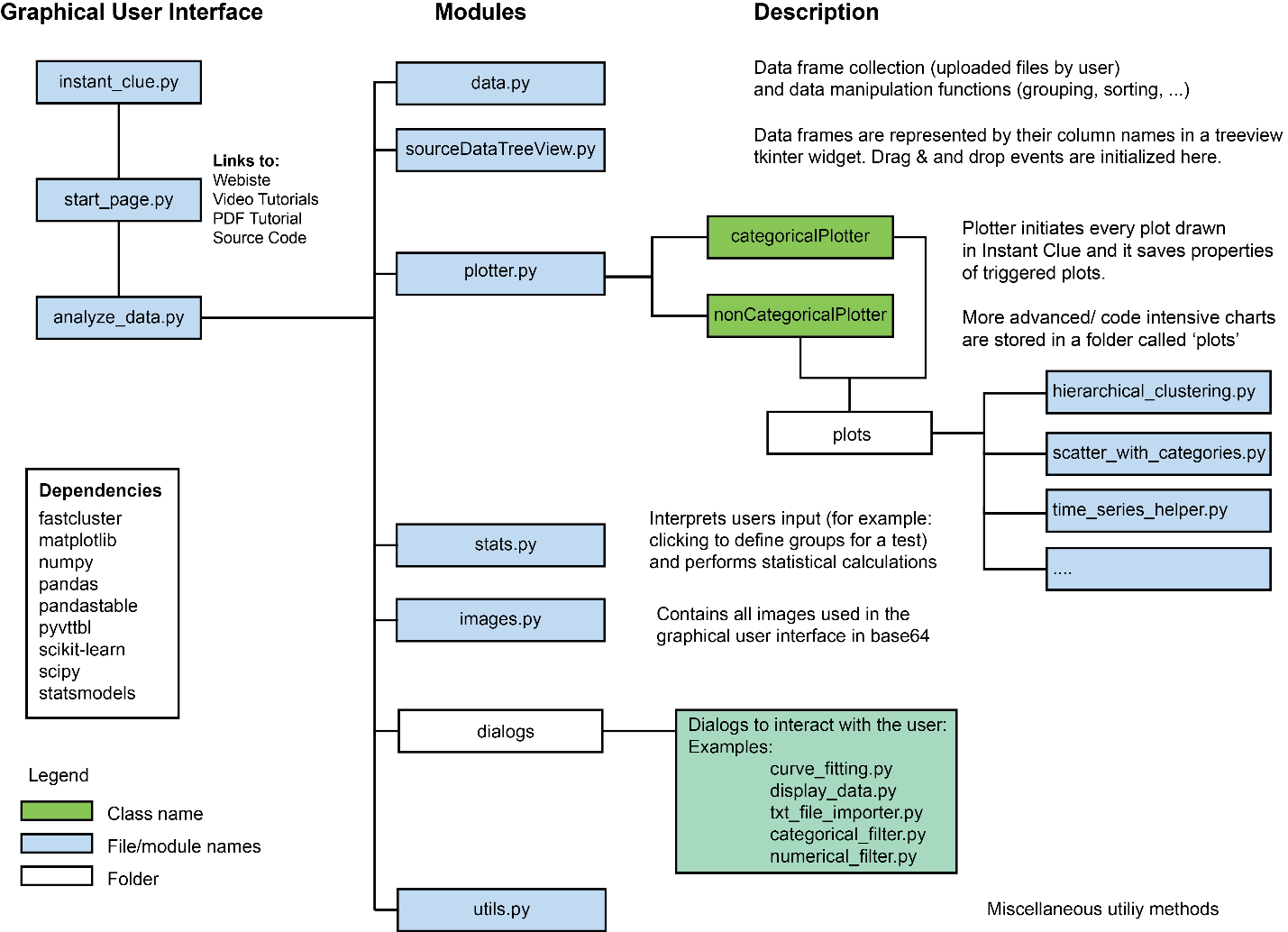
**

**Figure S2 - Architecture of Instant Clue.** The Graphical User (GUI) interface is built by three modules: instant_clue.py starts the application and displays the tkinter frame defined in start_page.py while analyze_data.py can be reached from the start_page which is a scaffold for the other modules handling data management, images, statistical calculations, drag and drop events and dialogs/popup to interact with the user. The source code is hosted on GitHub: <https://github.com/hnolCol/instantclue> and licensed under the GPL-3.

**
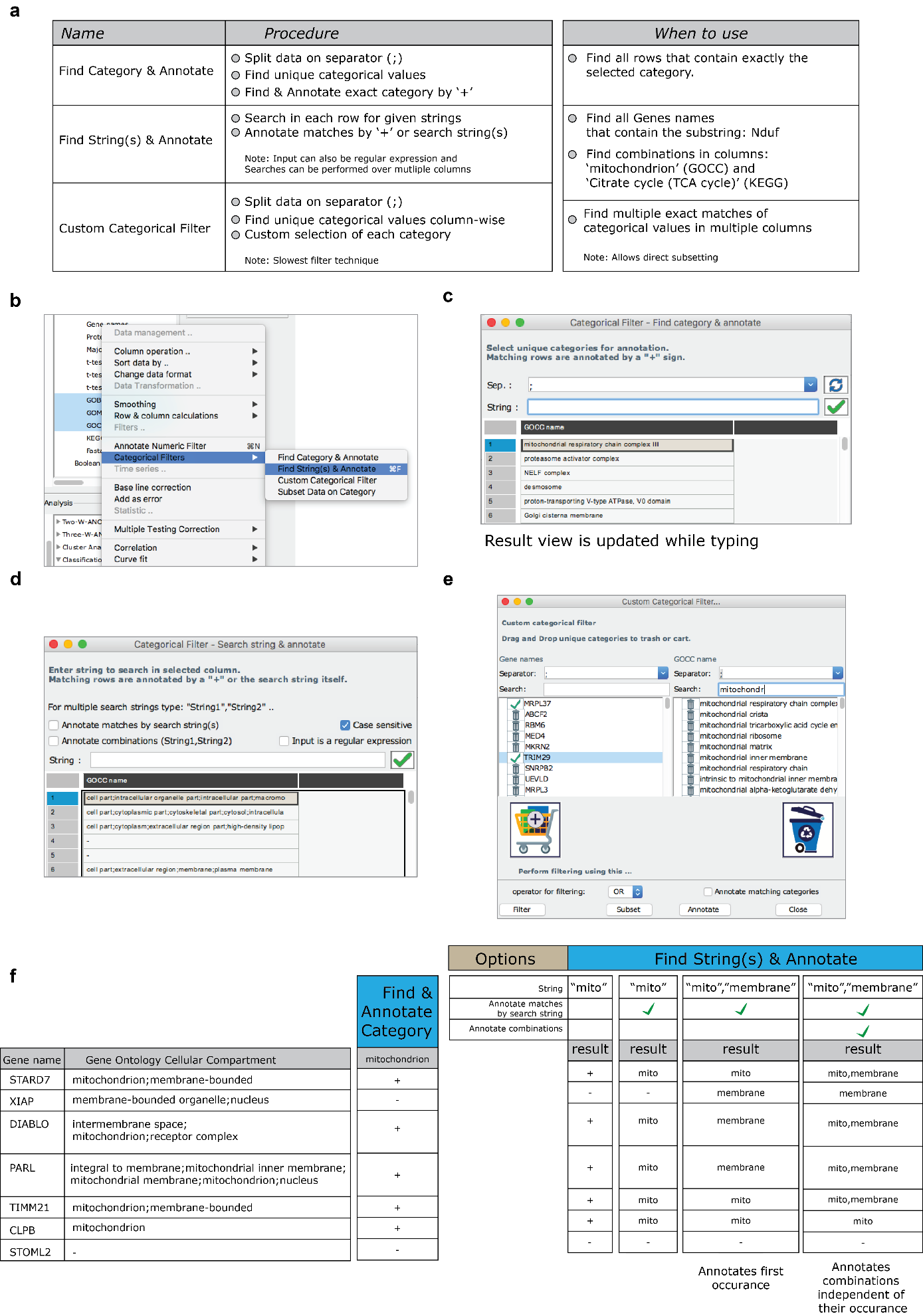
**

**Figure S3 Categorical Filtering in Instant Clue**. Categorical filter can be used to select a subset of the source data. In Instant Clue there are three different categorical filter which can be used for different purposes. The best way to explore the advantages of each filter is to apply them. In the following you will find a description of each filter using the “ExampleData02.txt” in the Example folder of the installation directory. a) Description and advantages of available categorical filters. b) Screenshot – drop-down menu. Categorical filters are applied on selected column. The filter “Find & Annotate Category” can only be applied on one column while all other can handle multiple columns. c) “Find Category & Annotate” d) “Search string(s) & Annotate” allows the user to find multiple strings. To search for multiple string the syntax is: “String1”,”String2”. It also accepts regular expressions by the user. e) “Custom Categorical Filter” – unique values are displayed for each column. Categories can be searched using the search input field, matching categories will be listed at the top of the list. The user can either select by double-click categories or by drag & drop of specific categories onto the cart or the trash icon. The filtering can either be applied using the OR or the AND operator. Three different options are available to apply the filter: i) Filter – deletes all non-matching rows, ii) Subset – creates a subset (new data frame) and adds it to the tree view. iii) Annotate – annotates rows that match the given criteria by ‘+’. If the “Annotate matching categories” is checked rows will be annotated by the selected categories instead of a ‘+’ sign. f) Table for demonstrating different settings in categorical filters. Columns: Gene names and Gene Ontology Cellular Compartment annotations (simplified). The results are displayed for result using the “Find & Annotate Category” filter selection the category “mitochondrion”. The “Find String(s) & Annotate” filter can be extended by two options: i) “Annotate matches by search string” – will annotate the search string that occurs first if option ii) “Annotate combinations” is not checked. If checked, combinations will be annotated independent of their occurrence. That means that in the given example a row will never be annotated by “membrane, mito”.


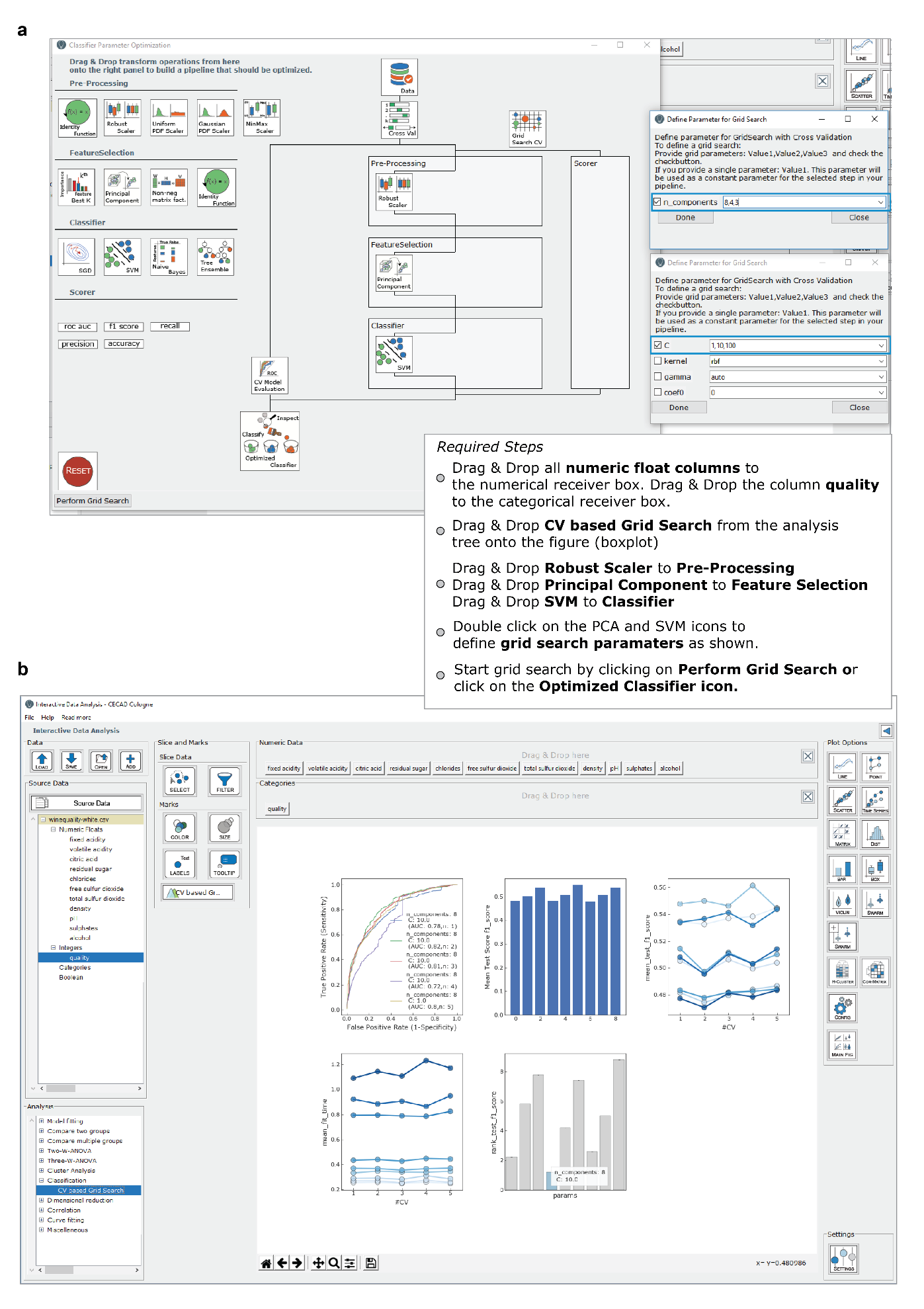
**Figure S4 Pipeline creation for cross-validated based grid search.** Supervised learning can be used to predict class membership of unseen data. Usually a prediction pipeline consists of three major steps: Pre-processing such as normalization (shown example: quantile normalization), Feature Selection – to reduce the number of features and save time, most important features can be selected or the number of features might be reduced by dimensional reduction, and the classification by an estimator/classifier. The steps can be optimized using a cross-validation based grid search. The general principal here is to use two cross validations: i) externally to use a subset for parameter evaluation (train) and then evaluating the best found estimator on the test data set (by default the number of splits is 5) and ii) internally to optimize parameter (e.g. train an estimator using a subset of the data (train) and test that estimator with a test dataset, 3 splits by default) (Figure 3 in main text). Noteworthy, the class distribution is kept equally between test and train datasets. This procedure can be computational intensive. The example shown uses the winequality-white.csv dataset in the installation directory. a) Dialog window to create a pipeline using the activities displayed on the left hand side by interactive drag & drop. Double-click on selected activities allows to modify constant parameters or to define the grid for parameter evaluation.

b) Results of a grid search in Instant Clue showing ROC curves (averaged for multiple classes) of the best estimator found. Legend indicates the optimized parameters. Mean test scores are displayed by a bar diagram using the different parameter settings as well as a point plot that indicates the mean test score in each cross validation split. Hovering over the charts will display a tooltip indicating the specific parameter settings. The middle-bottom panel shows the mean rank of the used settings. In this example the settings: number of components by PCA: 8 and parameter C (SVM): 10 were found to predict the classes within the outer test subset at the highest f1 score. For more information and explanations read the online tutorial. (http://www.instantclue.uni-koeln.de/tutorials.html) Since the scikit-learn python package is used the terminology is adapated and the package website is a great resource to learn more about classification task. At the moment only classification tasks not regression task (e.g. prediction a continues values instead of class) can be accomplished.

**
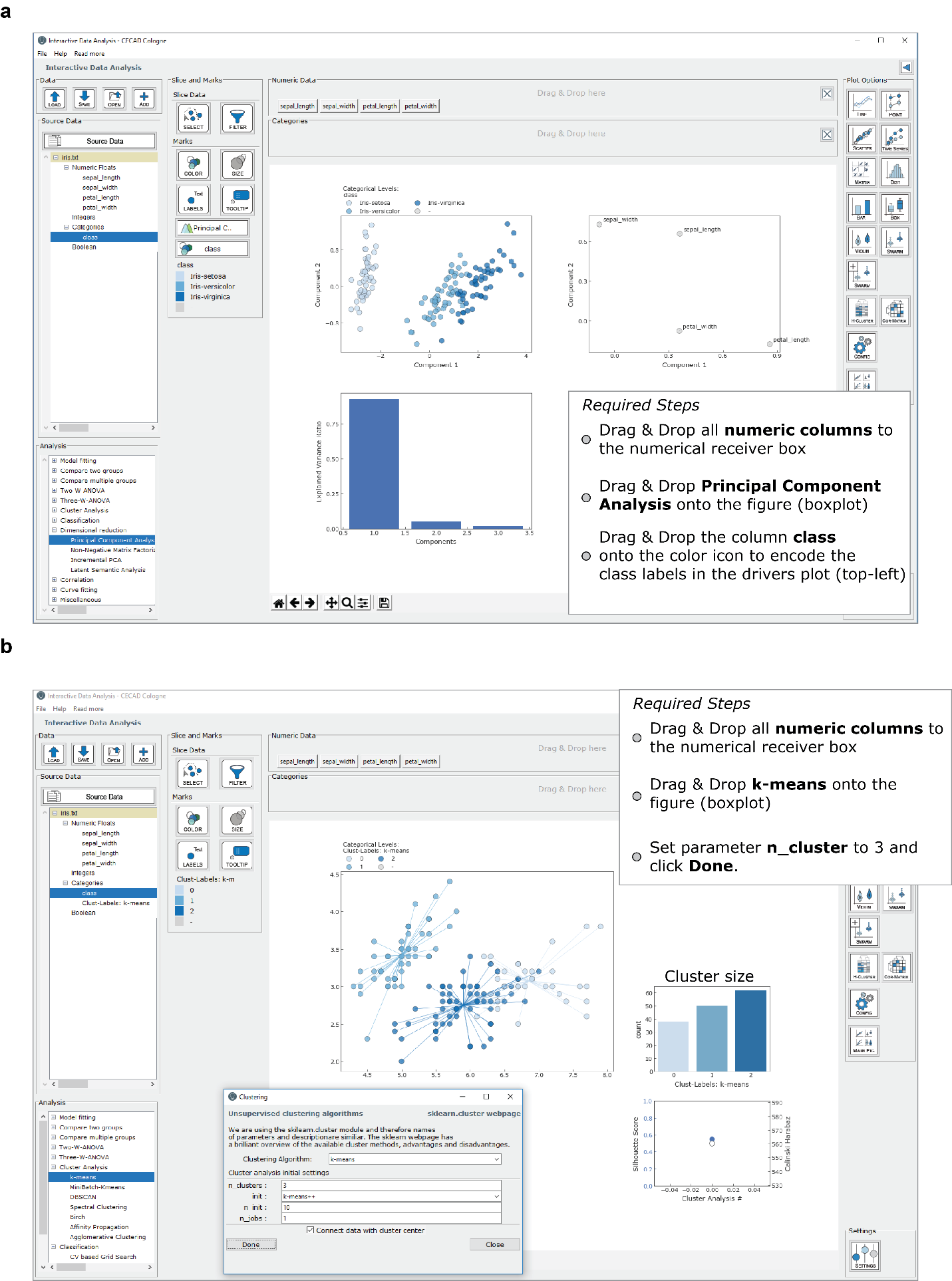
**

**Figure S5 – Principal Component Analysis and k-means clustering.**  Illustration using the Iris data set which is also included in the Instant Clue installation directory.

a) Screenshot of dimensional reduction (Principal component analysis). Three steps are required to reproduce the shown view and are shown in bottom-right corner: Use Drag & Drop to add the numerical columns: “sepal length/width” and “pedal length/width” to the numerical receiver box. A boxplot chart will appear. Apply dimensional reduction (PCA) by Drag & Drop of the activity: “Principal component analysis” onto the figure (boxplot). To color encode the class labels in the driver plot, drag & drop the categorical column “class” onto the “color” icon in the “Slice and Marks” frame.

b) Screenshot of a k-means clustering result. Use Drag & Drop to add the numerical columns: “sepal length/width” and “pedal length/width” to the numerical receiver box. A boxplot chart will appear. Drag & Drop the activity: “k-means” onto the figure. A dialog window will open that allows the user to set various parameters. The “n_cluster” parameter was set to 3 since the iris data set contains three different iris species. A detailed explanation for each subplot can be found in the online tutorial <https://www.instantclue.uni-koeln.de/tutorials.html>.

**
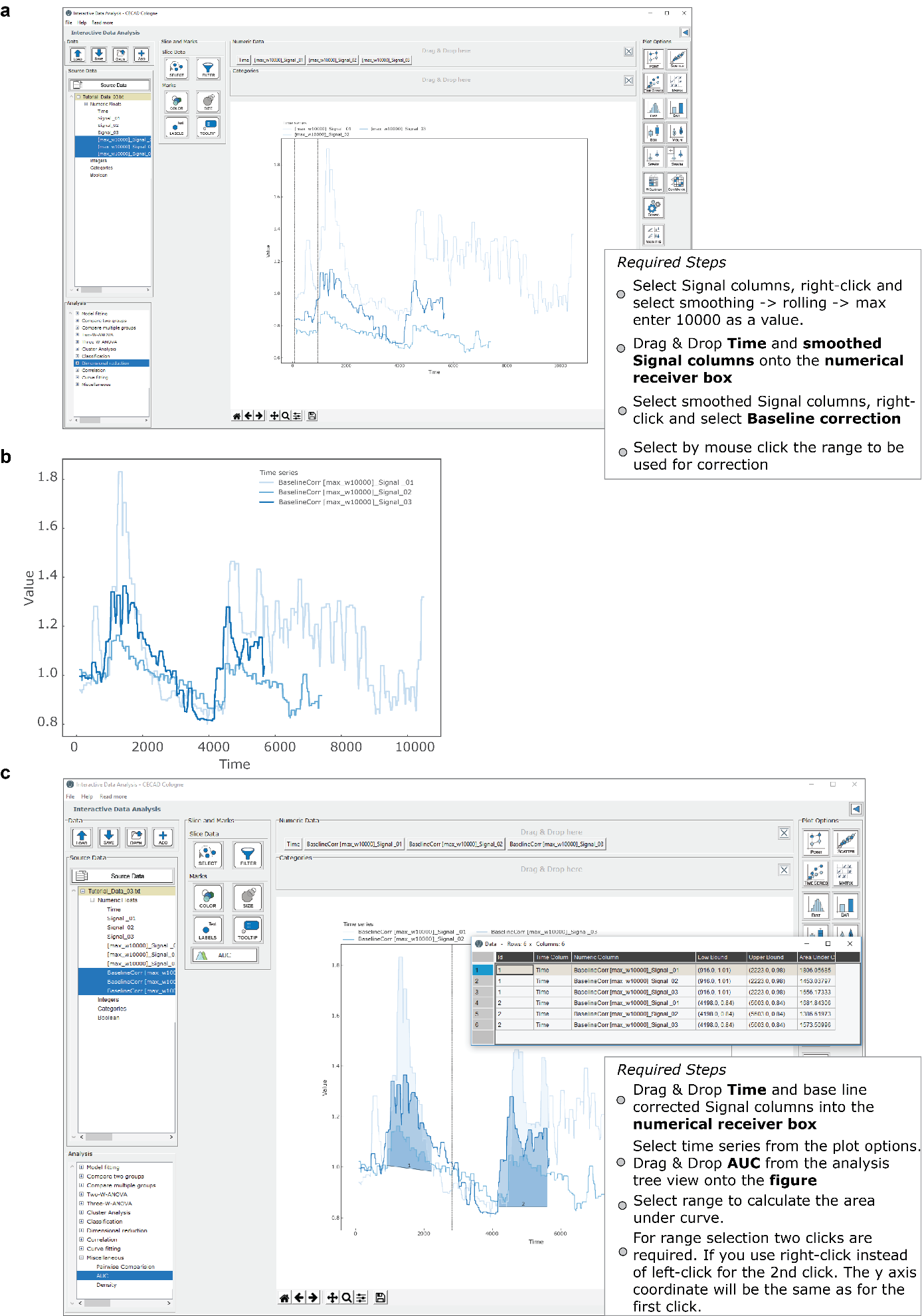
**

**Figure S6 Time series analysis.** Demonstration using the TutorialData03.txt file located in the Instant Clue installation directory. Required step for a) Baseline correction and b) Area under curve calculation are listed.


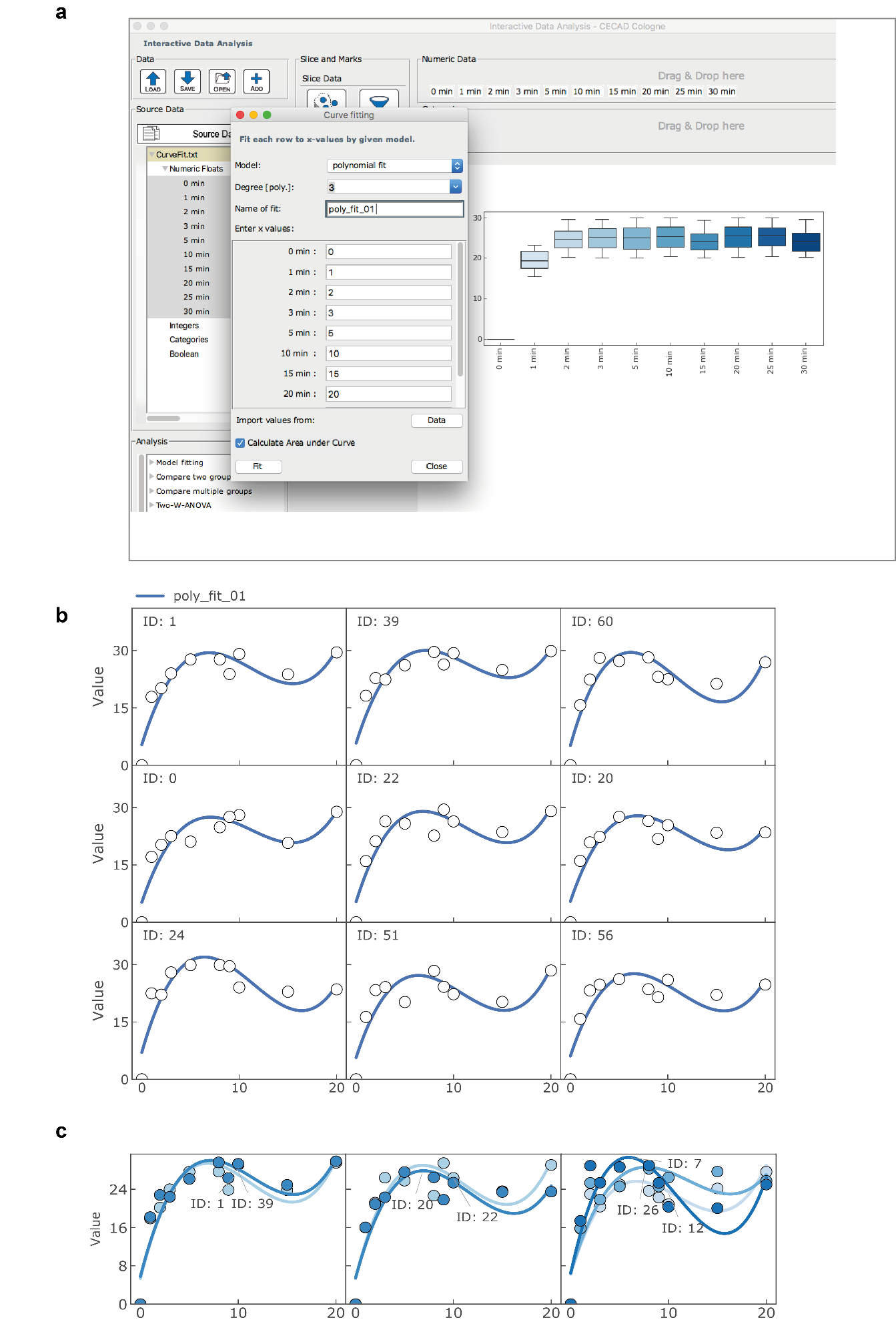


**Figure S7 – Curve fitting.**  a) Screenshot of curve fitting dialog. The data file curveFit.txt can be found in the installation directory and contains 10 randomly drawn amplitude values over a time period of 30 min. The x values, the curve fit name and the function to be used need to be defined by the user. Moreover, the area under curve (AUC) can be calculated if the check button is selected using the trapezoidal rule. Upon successful fitting, columns containing the fitted coefficients, squared r, as well as the area under curve values, are added to the data. Notably, all data that are needed to display performed curve fits are stored internally, meaning that the added columns can be deleted. b) Raw output of the activity (“Display curve fit(s)”) in Instant Clue. The number of subplots in the output can be chosen by the user. c) Multiple rows in the data can be combined in subplots in a customizable fashion. See the tutorial at <https://www.instantclue.uni-koeln.de/tutorials.html> for more information.
